# Food Safety and Adverse Selection in Rural Maize Markets

**DOI:** 10.1111/1477-9552.12350

**Published:** 2019-07-29

**Authors:** Didier Kadjo, Jacob Ricker-Gilbert, Gerald Shively, Tahirou Abdoulaye

**Affiliations:** 1 Didier Kadjo is Co-Manager, ONYX, Cote d’Ivoire; 2 Jacob Ricker-Gilbert and Gerald Shively are both in the Agricultural Economics Department, Purdue University, Indiana, USA; 3 Tahirou Abdoulaye is a Senior Agricultural Economist, IITA, Nigeria

**Keywords:** Adverse selection, asymmetric information, food safety, C13, D13, O12, O33, O39

## Abstract

Without enforced standards or reliable third-party verification, food safety threats such as pesticide residues and aflatoxin contamination are generally unobservable or only partially observable to both buyers and sellers, especially of staple foods in rural maize markets in sub-Saharan Africa. As a result, sellers have more information about food quality than do buyers. Such information asymmetries can impede market development and undermine human health. We study farm household behaviour in the context of imperfect food safety information. We pool observations obtained from 707 food storage containers maintained by 309 farm households in Benin, surveyed following the maize harvests of 2011/2012 and 2013/2014. Our results indicate that when a household perceives a food safety risk associated with application of insecticides, on average it is 33 percentage points less likely to apply insecticides to maize it intends to consume than it is to maize it intends to sell. These individuals are also more likely to sell maize than households without food safety concerns. Results highlight the potential value of improved storage technologies and quality control to promote market transactions and reduce hidden health risks.

## 1 Introduction

Do food safety concerns influence a farm household’s decision to apply storage chemicals? Do these factors affect the household’s decision to sell grain, thereby creating the potential for unsafe food to be sold in rural markets? In this article, we investigate these issues in Benin’s rural maize markets. At present, inconsistent and poor-quality grain is prevalent in many markets in Benin, a phenomenon widely observed elsewhere in developing countries, especially sub-Saharan Africa (e.g. Fafchamps, 2004; Hodges *et al*., 2011). Improving the quantity and quality of food is a fundamental component of efforts to modernise the food supply chain in low-income settings. Because low quality food is often associated with food safety concerns and potential health risks, the presence of low-quality grain also reduces the scope for smallholders to participate in international markets.

Given the range of relevant issues, it is somewhat surprising that improving food quality and safety has received relatively little attention in most countries of subSaharan Africa (SSA). In general, food quality consists of both observable and unobservable attributes. For cereals such as maize, observable quality attributes that may affect food safety include insect damage and mould. Buyers and sellers can typically assess these characteristics during market transactions. In Benin, for example, Kadjo *et al*. (2016) find that appropriate adjustments for observable reductions in quality are built into maize prices, either explicitly or implicitly. In contrast, unobservable attributes such as the presence of aflatoxins and other mycotoxins or chemical residues remain hidden to both buyers and sellers, and thus impede the development of food markets. They can also introduce serious health risks (Hoffmann and Gatobu, 2014; Hoffmann and Moser, 2017).

Since sellers know their storage practices, they potentially have more information than buyers about the unobservable attributes that affect the quality of food items offered for sale. This information asymmetry creates the potential for adverse selection (Akerlof, 1970; Fafchamps, 2004). For example, if buyers cannot observe pesticide residues themselves, cannot easily test for residues, and cannot obtain reliable information about grain quality from a third party, price adjustments based on quality are unlikely because buyers cannot readily differentiate between safe and unsafe grain. This reduces the returns to preserving high quality grain that is free from chemical insecticides and other contaminants and creates disincentives for farmers to market high quality food.^2^

Even though food safety concerns due to aflatoxin and pesticide residues are major problems with marketed maize in SSA, asymmetric information might not be a systematic characteristic of maize transactions in informal markets. In fact, many small-holders have limited knowledge of the potential harm chemical residues and/or aflatoxins may cause (Williamson *et al*., 2008; Fafchamps *et al*., 2008; Narrod *et al*., 2011; N’dede *et al*., 2012).^3^ As a result, they may not necessarily consider these attributes when spraying or drying their maize. In this context, asymmetric information about unobservable quality attributes depends on sellers’ subjective beliefs about the risk of food contamination from their storage practices.

Accordingly, our first objective is to investigate the quality of maize marketed by rural smallholder households in Benin. In doing so, we test two related hypotheses. Hypothesis 1 tests whether a household is equally likely to apply storage chemicals to maize that it intends to sell and maize that it intends to consume. Hypothesis 2 tests whether the probability of storage chemical application to maize a household intends to sell increases with its awareness of food safety risks.^4^ Our second objective is to evaluate whether, and to what extent, adverse selection occurs in the market. To do this, we test two additional hypotheses. Hypothesis 3 tests whether sales are related to expenditures on storage chemicals. Hypothesis 4 tests whether bad grain is driving out good grain from rural markets, in the sense that informed sellers (i.e. those who are aware of food safety risks) who use insecticides are more likely to sell maize than their uninformed counterparts.

We test these hypotheses using two-wave panel data collected after the 2011/2012 and 2013/2014 harvest seasons in Benin. We use a balanced panel of 309 households who were surveyed in each wave to analyse households’ market behaviour in the face of potential adverse selection. In our sample, some households reported multiple storage units, which they designated alternately for home-consumption and/or sales. This provides an opportunity to work with 707 ‘container-level’ observations to analyse how households apply chemical insecticide to specific units of grain, depending on intended use.

We recognise that a household’s subjective belief about the risk of food contamination creates potential endogeneity between the treatment and sales decisions. To address this issue, we use a Mundlak Chamberlin device (analogous to a fixed effects estimator in non-linear models) to control for unobservable household-level heterogeneity. We also account for other covariates that might be correlated with households’ perception about the risk of food contamination. We then conduct a series of robustness checks in which we compare our results under parsimonious regressions with fully specified regressions. To measure the perceived risk of food contamination we use two variables that capture a household’s attitude regarding consuming maize sprayed with chemical insecticide. The first is the number of days the household believes it must wait (or usually wait) between spraying and consuming maize in order to render it safe. The second is a binary variable equal to one if the household considers the presence of chemical on maize to be unsafe for consumption, and zero otherwise. To rid our estimation of any confounding effect and ensure that these two variables measure the risk of food safety, we use a set of covariates that determine the household’s knowledge about good storage practices as control variables. Although these steps are directed at correcting for potential endogeneity, we refrain from making strong causal inferences since we rely on observational data. Nevertheless, the findings should identify associations between a household’ subjective belief about the risk of food contamination and its decisions regarding market participation.

To date, there is limited understanding of how quality concerns affect market participation in informal food markets in developing countries. Arslan and Taylor (2009) found that rural households in Mexico prefer to produce and consume their own crop, even at a cost well above the market price. Fafchamps *et al*. (2008) showed that information about the type of application of pesticide and post-harvest treatment is not passed along the value chains in non-staple food markets in India. Consequently, growers receive no incentive regarding unobservable crops attributes and are only interested in agricultural practices that raise the quantity sold or improve observable characteristics. Hoffmann and Gatobu (2014) were the first to attempt to explain smallholder farm households’ limited market participation in staple markets through the asymmetric information that exists for unobservable quality attributes. They used an experimental auction in which households were offered the opportunity to sell their maize and then, the following day, the opportunity to buy it back or buy replacement maize from the market. They found that participating households placed a higher value on own-produced maize *vis-à-vis* maize sourced from markets. They attribute the observed difference in willingness-to-pay as reflective of food safety concerns, and the asymmetric information between buyers and sellers. Our study builds on Hoffmann and Gatobu (2014) by explicitly estimating and testing how adverse selection could arise in informal markets.

## 2 Maize Quality and Marketing in Benin

Maize is the main staple in Benin, as in many other parts of SSA. Maize production and storage practices differ across regions in Benin due to local consumption patterns and spatial differences in the comparative advantage of maize compared with other crops. For example, maize is produced for domestic consumption in the south and as a cash crop in the north, where it is viewed as an alternative to cotton as a source of cash income.

Access to improved storage technologies remains a pressing constraint in many countries in SSA. Maize producers in Benin continue to face substantial storage losses because there is no modern technology that is readily available to households to preserve stocks from pest damage over extended periods (ADA, 2010).^5^ Recommended chemical insecticides for stored maize such as Actellic and Sofagrain were promoted in Benin by projects that facilitated credit access and supply of these insecticides during the 1990s (Adegbola, 2010). Unfortunately, the implementation of these projects did not address other long-term constraints to adopting these new technologies, such as high costs and low availability (Adegbola, 2010). Likewise, modern storage technologies, such as hermetic bags and metal silos that effectively eliminate storage losses from insects without using chemicals have not been successfully promoted in Benin. As a result, at the time of our study many rural households still used traditional conservation measures, such as ash or neem, or field insecticides, especially those marketed for cotton, and other chemicals they believe appropriate to deal with pest damage (Adegbola, 2010; Hell *et al*., 2008; Williamson *et al*., 2008). Even though these inappropriate pesticides may protect maize stocks from pest damage, their widespread use among households raises serious health concerns. For instance, fatal cases of chemical poisoning by misuse of chemical insecticides were reported in the early 2000s in Benin (Williamson *et al*., 2008). Yet until recently, many farmers still sourced both field and storage chemicals from informal and non-certified sources (Adechian *et al*., 2015).

Most maize marketed in Benin passes through informal channels. The lack of formal quality control in these informal markets creates the potential for asymmetric information between market participants. In the absence of quality control, market participants implement their own practices to verify maize quality. For example, wholesale traders may sample a portion of maize they intend to purchase to check quality, using informal practices such as smelling grain to detect the odour, or biting grain to check for moisture.

## 3 Conceptual Framework

We follow Hoffmann and Gatobu (2014) to investigate how households’ efforts in improving maize quality during the harvest season affect their actual market behaviour during the post-harvest season. During the harvest season, households spend time and money to harvest (reap, thresh, winnow), dry, transport, sell and store maize. In the post-harvest season households consume, sell and/or purchase maize. As in Antle and Pingali (1994) and Liu and Huang (2013), we account for the fact that some storage practices such as the use of insecticides may yield both positive effects (e.g. reduced insect damage) and negative effects (e.g. chemical contamination that could pose a health risk). To explain market participation behaviour we expand on Hoffmann and Gatobu (2014) by emphasising how a household’s subjective belief about unobservable quality attributes could influence market participation during the post-harvest season.

We consider a non-separable farm household model where a risk averse household with demographic characteristics ***Z*** maximises its utility during the post-harvest season from consuming (superscript *c*) a staple food $\left[q_{h}^{c}\left(a\right)\right]$ sourced from their own production (subscript *h*) or purchased (subscript *p*) from market $\left[q_{p}^{c}\left(b\right)\right]$ where *q* represents maize quantity, *a* and *b* represent attributes that can be either good unobservable ($\overset{¯}{u}$) or bad unobservable (*u*) traits.^6^ The household also obtains utility from consuming a non-food good (*x*).

We can state the household’s maximisation problem as follows:

(1) $$\text{Max }U\left(q_{h}^{c}\left(a\right)+q_{p}^{c}\left(b\right),x;Z\right).$$

Quality attributes are increasing in a set of storage practices such as the use of storage inputs (*n*), and labour time (*l*) allocated to practices such as drying. A household may adopt these practices to achieve positive quality attributes ($\overset{¯}{u}$) while at the same time generating negative attributes (*u*).^7^ We assume the household forms a subjective assessment about the food safety of maize at the time of storage. Let *ρ* represent the probability that a household suspects that the negative attribute could be harmful. We can write the expected quality for unobservable (*u*) such as:

(2) $$E\left(u\right)=u^{E}=\rho \left(\underset{¯}{u}\right)+\left(1-\rho \right)\overset{¯}{u}.$$

We can also write the negative attribute as a relative rate of chemical contamination in safe maize such that $\underline{u}=\tau \bar{u}$ with the non-zero parameter *τ* being <1. If *τ* = 1 then there is no residue, so that safe maize is obtained. Equation (2) becomes:

(3) $$E\left(u\right)=u^{E}=\rho \tau \overset{¯}{u}+\left(1-\rho \right)\overset{¯}{u}=\left[(1-\left(1-\tau \right)\rho \right]\left(\overset{¯}{u}\right)=\left(1-\rho \prime \right)\left(\overset{¯}{u}\right).$$

Equation (3) shows that under a risk of food contamination the household knows that good unobservable characteristics may not be achieved because of the potential risk of chemical residues.

Furthermore, let us assume a situation where the household achieves good maize quality ($\overset{¯}{u}$). The household allocates its stock (*Q*) to consumption $\left(q_{h}^{c}\right)$ or sales $\left(q_{h}^{m}\right)$ as shown in equation (4); the superscripts (*c*) and (*m*) representing consumption and sales, respectively.

(4) $$Q=q_{h}^{c}\left(\overset{¯}{u}\right)+q_{h}^{m}\left(\overset{¯}{u}\right).$$

Equation (5) is the income balance:

(5) $$p.q-p_{n}n\left({\overset{¯}{u}}^{E}\right)-wl\left({\overset{¯}{u}}^{E}\right)+p_{h}^{m}\left(\overset{¯}{u}\right)q_{h}^{m}\left(\overset{¯}{u}\right)\geq p_{p}^{c}\left(b\right)p_{p}^{c}\left(b\right)+x.$$

The parameters *p* and *q* represent the price and the quantity of maize sold during the harvest season, respectively, while the parameters *p_n_* and *w* denote the price for insecticide storage, and the labour wage, respectively. The price for the non-food good, *x*, is normalised to one. A household invests in insecticides and labour during the harvest season because it expects to achieve a certain maize quality in the post-harvest season, represented by ${\overset{¯}{u}}^{E}$. In equation (5) the parameter $p_{h}^{m}$ is the price a household receives for selling maize during the post-harvest season, whereas $p_{p}^{c}$ is the price they pay for purchasing maize from the local informal market.

We consider an alternative situation where a household wants to decide how to choose the amount of maize to sell when that maize could have been allocated to consumption.^8^ Thus, maize sales become an endogenous variable.

If the households are indifferent to quality, then simple first-order conditions with respect to the endogenous variables $q_{h}^{m}$, $p_{p}^{c}$, and *x* leads to the standard result of the ratio of marginal utility equates to the price ratio.

Specifically, the first-order condition with respect to the amount of maize sold is:

(6) $$\frac{\partial ℒ}{\partial q_{h}^{m}}\equiv -U_{q_{h}^{m}}+\lambda q_{h}^{m}\left(\overset{¯}{u}\right)=0.$$

The parameter λ is the Lagrange multiplier for equation (5). The parameter marginal $U_{q_{h}^{m}}$ is the utility with respect to the amount of maize sold.

If the household is concerned about maize quality, we can extend the maximisation problem, adding an additional endogenous variable for quality, $\overset{¯}{u}$. The new marginal utility for consuming maize with a given quality attribute will increase more than before due to the link between quality and quantity. In other words, marginal utility with respect to maize quality increases because marginal utility is increasing in maize quantity, and maize quantity is in turn, increasing in maize quality.^9^ The first-order condition with respect to maize quality is such that:

(7) $$\frac{\partial ℒ}{\partial \overset{¯}{u}}\equiv U_{q_{h}^{m}}\frac{\partial q_{h}^{m}}{\partial \overset{¯}{u}}+\lambda \left[p_{h}^{m}\left(\overset{¯}{u}\right)\left(\frac{\partial q_{h}^{m}}{\partial \overset{¯}{u}}\right)+q_{h}^{m}\left(\overset{¯}{u}\right)\frac{\partial p}{\partial \overset{¯}{u}}-p_{n}\frac{\partial n}{\partial \overset{¯}{u}}-w\frac{\partial l}{\partial \overset{¯}{u}}\right]=0.$$

When we substitute equation (6) in equation (7), we can rewrite equation (7) as:

(8) $$\frac{\partial ℒ}{\partial \overset{¯}{u}}\equiv \lambda_{p_{h}^{m}}\left(\overset{¯}{u}\right)\frac{\partial q_{h}^{m}}{\partial \overset{¯}{u}}+\lambda \left[p_{h}^{m}\left(\overset{¯}{u}\right)\left(\frac{\partial q_{h}^{m}}{\partial \overset{¯}{u}}\right)+q_{h}^{m}\left(\overset{¯}{u}\right)\frac{\partial p}{\partial \overset{¯}{u}}-p_{n}\frac{\partial n}{\partial \overset{¯}{u}}-w\frac{\partial l}{\partial \overset{¯}{u}}\right]=0.$$

We can also rewrite equation (8) to obtain equilibrium, as shown in equation (9):

(9) $$2p_{h}^{m}\left(\overset{¯}{u}\right)\frac{\partial q_{h}^{m}}{\partial \overset{¯}{u}}+q_{h}^{m}\left(\overset{¯}{u}\right)\frac{\partial p}{\partial \overset{¯}{u}}=p_{n}\frac{\partial n}{\partial \overset{¯}{u}}+w\frac{\partial l}{\partial \overset{¯}{u}}.$$

The left-hand side of equation (9) is the marginal value of the investment in quality preservation. The right-hand side represents the marginal cost of the efforts invested in quality preservation. Equation (9) indicates that the marginal cost of the efforts to obtain good quality is equal to the sum of (i) the value gain from the additional amount of maize due to the quality improvement, and (ii) the price premium for quality in the market.^10^ The value gain from the additional amount of maize is achieved if the household decides to sell this additional amount obtained from quality improvement into markets. By contrast, price premium depends on market characteristics. Thus, if there is little if any price premium, as we may assume given the current market structure in many sub-Saharan markets, efforts may be costlier than the market will pay for. Consequently, a rural household may not find it optimal to allocate a large amount of maize if any with good quality attributes $\overset{¯}{\left(u\right)}$ to markets. This is more so in a situation where uncertainty of the quality of maize to purchase from markets is high.

However, equation (9) has somewhat different implications if a household believes the use of a chemical insecticide might be unsafe, i.e. a situation where the probability of risk of residue is different from zero. Consequently, we obtain a new form of the chemical use equation where the decision depends on expected attributes. Assuming a linear form of a household’s decision to use an input (chemical) based on expected quality, we can write the marginal effect of chemical use under the new form (*n_f_*) that accounts for a contamination risk as a function of the initial chemical use without risk such that:

(10) $$\frac{{\partial n}_{f}\left(\overset{¯}{u}\right)}{\partial \overset{¯}{u}}=\left(1-\rho \prime \right)\frac{\partial n\left(\overset{¯}{u}\right)}{\partial \overset{¯}{u}}$$

Equation (10) implies that we can derive a general form for equation (9) that includes a household’s subjective belief about maize safety from storage practices such as insecticide use.

We obtain the following equilibrium condition:

(11) $$2p_{h}^{m}\left(\overset{¯}{u}\right)\frac{\partial q_{h}^{m}}{\partial \overset{¯}{u}}+q_{h}^{m}\left(\overset{¯}{u}\right)\times \frac{\partial p}{\partial \overset{¯}{u}}=\left(1-p\prime \right)p_{n}\frac{\partial n}{\partial \overset{¯}{u}}+w\frac{\partial l}{\partial \overset{¯}{u}}$$

Equation (11) implies that the presence of the parameter (1 − *ρ*′), which is ≤1, may reduce the marginal cost of investment in quality improvement (right-hand side). Therefore, we argue that the marginal cost of investment in quality improvement or the reservation value for consuming grain quality from home-grown maize is lower under a perceived risk of food contamination (0 < *ρ*′ < 1) than otherwise (*ρ*′ = 0). This new equilibrium implies that a seller who suspects a risk of food safety could sell more maize into markets than an unsuspecting seller. The subsequent empirical estimation investigates whether this prediction applies to rural transactions in Benin’s maize markets.

## 4 Empirical Strategy

### 4.1 Testing Hypotheses 1 and 2

Our first objective is to identify which maize quality a household will most likely sell into markets. Equation (12a) represents a binary response model in which the dependent variable, is equal to one if a household *i* applies chemical insecticides (*Chem_ijt_*) to maize stored in container *j* during the agricultural year *t*, and equals zero otherwise:

(12a) $$P\left({\text{Chem}}_{\text{ijt}}=1|\text{Sal},\text{Unsafe},X\right)=\Phi \left(\alpha_{0}+\alpha_{1}{\text{Sale}}_{\text{ijt}}+\alpha_{2}{\text{Unsafe}}_{\text{it}}+X\alpha_{3}+a_{i}+u_{\text{ijt}}\right).$$

*P*(.) indicates probability, Φ is the standard normal cumulative distribution function. The variable *Sale_ijt_* denotes the household’s intended storage goal for the container of maize. The goals are either (i) home consumption only, (ii) sale only, or (iii) both home consumption and sale. We use consumption as the base storage goal, and the parameter α_1_ denotes the corresponding coefficient estimate for the variable *Sale_ijt_*. When the variable *Sale_ijt_* equals one for sale only, the coefficient α_1_ tests Hypothesis (1) that a household’s goal for sale has no statistically significant effect on its decision to apply insecticide to maize intended for sale compared to maize held in a container that is stored for home consumption.

The variable *Unsafe_jt_* denotes a household’s subjective belief about the risk of food contamination from the use of insecticide. It alsoreflects the level of asymmetric information about maize safety that buyers are aware of. To measure the risk of food safety we use two variables by emphasising the household’s attitude with respect to consuming maize sprayed with chemical insecticides. The first is a continuous variable that mimics the probability of a risk of chemical residues. We asked households the number of days they believe they must wait (or they usually wait) between spraying and consuming maize to render it safe. We call this variable the latency period or the number of days for maize safety.^11^ We assume that the longer latency period, the greater is a household’s subjective belief about risk of food contamination. The second is a binary variable constructed by asking households whether they would consume maize treated with chemical insecticides themselves. It takes a value equal to one if a household considers the presence of chemicals on maize to be unsafe and therefore inappropriate for consumption irrespective of any supposedly risk preventing practices such as soaking or drying grain. To rid our estimation of any confounding effect and ensure that these two variables only pick up the risk of food safety, we carefully net out the effect of knowledge by controlling for variables that represent a household’s potential sources of information about recommended storage practices. The vector ***X*** comprises these variables and other covariates such as the number and the type of storage containers. The parameter *a_i_* represents household-level unobserved heterogeneity whereas the parameter *u_ijt_* is the idiosyncratic error.

We modify equation (12a) to investigate how a household applies insecticide to maize held for sales when it perceives a risk of food contamination as opposed to a situation where no risk is perceived. To do so we include an interaction term between a household’s storage goal and its subjective belief about the risk of food contamination as specified in equation (12b):

(12b) $$\begin{array}{l} P(Chem_{ijt}=1|Sal,Unsafe,X)\\ \;=\Phi (\beta_{0}+\beta_{1}Sal_{ijt}+\beta_{2}Unsafe_{it}+\beta_{3}Sal_{ijt}\times Unsafe_{it}+X\beta_{4}+a_{i}+u_{ijt})\cdot \end{array}$$

With the presence of the interaction term, we can further investigate the potential risk of adverse selection by assessing which maize quality is most likely sold into markets. The supposedly ‘good-quality grain’ is a maize stock intended for sale that a household unaware of the risk of food contamination would spray with chemical insecticide. The estimate and the statistical significance of the coefficient *β*_1_ evaluates how likely an uninformed household is to spray this type of maize compared to home-consumed maize. Conversely, ‘bad-quality grain’ is maize for sale that a household who is aware of risk of food contamination sprays with chemical insecticide. The joint estimate and the statistical significance of the coefficients *β*_1_ and *β*_3_ test Hypothesis 2, namely that the probability that a household that is aware of the risk posed by applying chemical insecticides to maize would more likely spray grain intended for sale than grain intended for home consumption. A positive coefficient *β*_3_ would suggest that the more a household perceives a food safety concern from using insecticides, the more likely it will be to apply chemical insecticides on a container intended for uses other than consumption (i.e. sales, or sale & consumption).^12^

### 4.2 Testing Hypotheses 3 and 4

Our second objective is to estimate how a household’s investment in chemical insecticides affects the quantity of maize it sells during the post-harvest season. A logical assumption is that the container-level behaviour we previously investigated might determine actual market transactions. However, we are unable to identify the quantity of maize sold that is initially sourced from a given storage container. Therefore, we analyse market transactions at the household level for Hypotheses 3 and 4, while accounting for the key determining variables of the container-level behaviour.

We consider a household’s market participation decision to consist of two independent decisions, the decision to sell maize and the quantity to sell. Our interest is to analyse how investments in storage technologies and practices drive the quantity to sell. Equation (13a) specifies a linear model of the factors that affect the quantity of maize sold into markets conditioned on the decision to sell as follows:

(13a) $$Q_{it}=\theta_{1}{\text{Exp}}_{it}+\theta_{2}{\text{Unsafe}}_{it}+Z_{it}\theta_{3}+b_{i}+\varepsilon_{it}.$$

where the variable *Q_it_* represents the kilograms of maize sold in the market by the household *i* during the post-harvest season *t*, estimated in log form. *Exp_it_* represents households’ expenditure on storage chemical insecticides. The estimate and the statistical significance of the coefficient θ_1_ tests Hypothesis 3: that investments in insecticides have no statistically significant effect on the amount of maize that a household sells in the market. The vector ***Z*** comprises households’ characteristics and market variables. It also includes container-level variables such as households’ storage goal and the number of storage technologies. The parameter *b_i_* is a household-level fixed effect to account for unobserved heterogeneity, and the parameter *ε_it_* is the idiosyncratic error. The variable *Unsafe_it_* is defined as before.

To evaluate how households’ beliefs about food safety and quality affect the decision to sell, we specify equation (13b), which adds to equation (13a) an interaction term between expenditures on chemical insecticides and households’ subjective belief about the risk of food contamination:

(13b) $$Q_{it}=\delta_{1}{\text{Exp}}_{it}+\delta_{2}{\text{Unsafe}}_{it}+\delta_{3}{\text{Exp}}_{it}\times {\text{Unsafe}}_{it}+Z_{it}\delta_{4}+b_{i}+\varepsilon_{it}.$$

The presence of the interaction term contrasts the effect on sales of a household’s investments in insecticides under two risk situations. We cantest the presence of adverse selection in maize markets by assessing whether ‘bad quality’ sellers trade the most into markets. The supposedly ‘good-quality sellers’ are in fact uninformed households who spend on chemical insecticides, but do not actually suspect the risk of food contamination. We evaluate the effect of this category of households on maize sales through the estimate and the statistical significance of the coefficient *δ*_1_. In contrast, ‘bad-quality sellers’ are those who spend on chemical insecticides while suspecting the risk of food contamination. We evaluate the effect of this category on market transactions through the joint estimate and statistical significance of the coefficients *δ*_1_ and *δ*_3_. Thus, a positive and statistical significant coefficient *δ*_3_ tests Hypothesis 4, that ‘bad quality sellers’ sell more of their stocks than ‘uninformed sellers’ do.^13^

## 5 Identification Strategy

### 5.1 Omitted variable bias

The use of panel data in this context allows us to address the issue of unobserved heterogeneity. Since we estimate equations (12a) and (12b) as binary response models via probit, the use of fixed effects could result in inconsistent parameters when applied to non-linear models due to the incidental parameter problem (Wooldridge, 2010). We circumvent this issue by using the Mundlack-Chamberlin (MC) device that deals with unobserved heterogeneity, denoted as *a_i_* in non-linear models (Mundlak, 1978; Chamberlain, 1984). Using the MC-device, the assumption of independence between covariates and unobserved heterogeneity can be relaxed by modeling *a_i_* in a linear form as follows:

(14) $$a_{i}=\varphi_{ij}+{\overset{¯}{W}}_{i}\xi +e_{it}.$$

The MC device assumes that $e_{it}|{\overset{¯}{X}}_{it}\sim$ *Normal* $\left(0,\sigma_{i}^{2}\right)$ where $\overset{¯}{W}$ is the household time average of all time-varying covariates in equations (12a) and (12b). This specification provides estimates that are analogous to household FE (within) estimation (Wooldridge, 2010). The MC device is also appropriate for dealing with unobserved heterogeneity in equations (13a) and (13b).^14^

In addition, we deal with potential omitted variable bias for a household’s storage goal in equations (12a) and (12b), as one could argue that this variable is not random. In this application storage goal is measured at the harvest period and therefore before the household applies chemical insecticide to a storage container. Moreover, we believe that accounting for households’ physical and liquidity assets in equations (12a) and (12b) could address any omitted variable bias issues. Indeed, it is well known that credit and assets are constraints to market participation in rural areas (Carter and Barrett, 2006; Barrett, 2008; Stephens and Barrett, 2011). These factors could also affect the household’s ability to acquire chemical insecticides. In addition, we evaluate the robustness of our estimate by comparing a parsimonious estimation to a full regression.

Because we define the use of chemical insecticides as an endogenous variable in equations (12a) and (12b), we also deal with potential omitted variable bias for the covariate expenditures on insecticides in the model of market participation (equations (13a) and (13b)). We assume that a household’s expenditures on insecticides during the harvest season determine its market participation later in the post-harvest season. To avoid omitted variable bias from variables that determine both the container-level behaviour and market participation, we control for covariates such as households’ storage goal, the number of storage containers used in the household, the quantity of maize stored in the household and the type of chemical insecticide. In addition, we consider factors that reflect how easily a household may obtain chemical insecticides and market information. Thus, we incorporate the distance from home to the main market, the presence of an extension agent in the village and the presence of an input dealer in the village as additional control variables. Another issue could be that access to pesticides may not be random. Studies and field observations reveal that many households face constraints to accessing appropriate storage chemical technology. Because access to certified chemical insecticides is limited, many households use whichever pesticide they believe appropriate to deal with insect damage. They may have access to farm pesticide from any available input dealer in the village or in the markets (Ricker-Gilbert and Jones, 2015).^15^ More generally, we hypothesise that controlling for covariates that account for market participation and access to storage insecticides may take care of other possible endogeneity of chemical use caused by omitted variable bias. Here again, we evaluate the robustness of our estimate by comparing a parsimonious regression to a full regression.

We also address the potential endogeneity of a household’s subjective belief about the risk of food contamination in equations (12a), (12b), (13a) and (13b). This covariate might be endogenous, because it most likely depends on a household’s awareness about the risk of food contamination from the use of chemical insecticides. In each empirical model, we control for variables that could represent for a household, potential sources of information about chemical use that also affect the decision to apply insecticide to a maize container or to sell maize. For example, we account for whether the household’s head has received any information about how to use chemical insecticides and its impairment effect. We consider a household’s experience from any reported cases of chemical intoxication in the community. We also believe that characteristics such as the education level of the household’s head and the number of children in school could influence how the household perceives the risk of chemical contamination (Mabe *et al.*, 2017). Furthermore, we use two alternative variables to determine the directional effect of a household’s subjective belief about the risk of food safety.

### 5.2 Reverse causality and simultaneity bias

We address the issues of simultaneity and reverse causality between the tested covariates and the dependent variables by suggesting similar arguments. In equations (12a) and (12b), we ask households what was their storage goal at the time they were about to store maize. This assumes that they knew their storage goal before storing and then applied insecticides to maize stored in a container. Similarly, in the model of market participation (equations (13a) and (13b)), expenditure on chemical insecticides also precedes the quantity sold into markets. In fact, many households purchase field insecticides during the planting season that they later use as chemical insecticides in storage. Otherwise, they acquire chemical insecticides during the harvest season or early post-harvest period to apply on maize stocks to be sold later in the season.

It could be argued that a household’s subjective belief about the risk of food contamination at the time of the survey may reflect its experience of using chemical insecticides. In other words, previous use of insecticides could have determined a household’s perception about the risk of food contamination. However, we treat a household’s subjective belief about the risk of food contamination as time-invariant in this application supposing that their perception is determined outside the time variant use of chemical insecticides. In fact, there is little change in most households’ storage practices between the 2 years of our panel survey, which might imply that households’ subjective belief remains unchanged over time. Because the household’s subjective beliefs are formed before the decision to use chemical insecticide on grain stocks and to sell maize, simultaneity bias should also not be an issue.

### 5.3 Corner solutions

Nearly 20% of households do not participate in maize sales transactions during the post-harvest season. Our tested covariates that drive a household’s decision to participate in markets are different from the ones that affect the quantity sold (see online Appendix B).

## 6 Data

### 6.1 Data collection

We use data from a survey conducted in 6 of the 12 departments in Benin. We first considered the three regions in Benin: North, Centre and South. In each region, we used reported maize yield to select the 50^th^ percentile of the most productive areas among the departments. However, we retained one department in the North, Toucoutouna, based on the prevalence of food insecurity, even though it was not among the most productive areas. The other steps of the survey to identify the households were random. Two districts were randomly chosen within a given department. Counties, called ‘Commune’, were also randomly selected in the district, followed by a random choice of villages. In the first stage, survey enumerators conducted a census of maize households in each of the 12 villages to identify the pool of households who produced maize. In the second stage, 30 households were randomly chosen among these households. Each person interviewed was the head of the household.

The survey covered a consumption cycle for each household for the two waves of data collection, namely 2011/2012 and 2013/2014 harvest seasons. The first wave of data cover 360 households, but only 309 of these households were successfully interviewed during the second wave or had a complete set of information.^16^ We end up with a balanced sample of 309 households and 618 individual observations in the balanced panel. But the sample represents an unbalanced panel of 707 observations when we pool data over storage containers and year.

Attrition is a potential concern. Of 360 households interviewed in the first wave of data collection, only 314 were successfully interviewed during the second round and, of these, 309 had complete information. We rely on these 309 households for our balanced panel. Unfortunately, there is no regression-based test for attrition bias in a two-period Correlated Random Effect (CRE). The regression models in our analysis control for attrition bias to the extent that attrition is related to the observed covariates and/or time-constant, unobserved effects (Mason and Ricker-Gilbert, 2013). In addition, none of the tested covariates explains a household’s probability of being re-interviewed during the second wave (see online Appendix C). As a result, we do not believe attrition bias is likely to arise in this analysis. For the household level analysis, we weight our sample by the inverse probability of selection to account for the probability that the household was randomly sampled for interview. Nevertheless, given our relatively small sample, we make no strong claims regarding the representativeness of our findings for Benin as a whole.

The survey focused on households’ storage practices during the harvest season and their market participation during the post-harvest season. Data were collected at the end of post-harvest seasons or at the early start of a new agricultural cycle.

### 6.2 Descriptive statistics

We asked households their storage goal at the time they were about to store maize. More precisely, we recorded the intended use and storage practices for each main container in a household. The pooled sample, over storage containers and across survey waves, represents 707 observations. Table 1 shows that 529 containers are from households who use only one container, of which 204 (39%) containers are used for consumption only, 23 (4%) for sales only, and 302 (57%) for both consumption and sales. Table 1 also indicates that 178 containers are from households who use more than one container. Among these containers, 12 (7%) are used for consumption only, 32 (18%) for sales only and 134 (75%) for both consumption and sales. Thus, for the pooled sample of 707 observations we end up with 30% of containers intended for consumption only, 8% for sales only and 62% for both consumption and sales.

**Table 1:** Application of chemical insecticide to maize stored in containers

	Full sample	=1 if HH uses chemical insecticide	
	(No. of containers)	(No. of containers)	(No. days of latency)
=1 if HH uses 1 container			
Consumption only	204	35	38
Sale only	23	5	34
Consumption & sale	302	66	37
Total	529	106	37
*P-value*		*(0.58)*	*(0.59)*
=1 if HH uses more than 1 container			
Consumption only	12	12	46
Sale only	32	32	50
Consumption & sale	134	32	49
Total	178	76	48
*P-value*		*n/a*	*(0.38)*
Full sample – container level			
Consumption only	216	47*	40
Sale only	55	37*	47
Consumption & sale	436	98	41
Total	707	182	42
*P-value*		*(0.00)*	*(0.19)*

***Notes:*** HH stands for household; *P-value* for *t*-test (or Chi-square for discrete variables) for the difference between maize containers intended for sale and maize containers intended for consumption: *P < 0.01; n/a = not applicable.

Table 1 shows that households decide to apply chemical insecticide to maize containers based on how they intend to use them. They apply insecticides to 182 (25%) out of 707 storage containers. However, only 20% of the chemicals sprayed on maize containers were certified for such use (e.g. Actellic and Sofagrain). The remaining 80% consisted of unidentified surplus farm insecticides. For the full sample, the percentage of containers with insecticides applied to them is much higher for storage containers used to store maize intended for sales (37 out of 55, i.e. 67%) compared to containers intended for consumption only (47 out of 216, i.e. 22%) and containers intended for both consumption and sales (98 out of 436, i.e. 22%).

We obtain additional insights when we account for households’ subjective beliefs about the risk of food contamination. In the full sample, households who kept container content for sales only, believe that 47 days of latency is needed on average compared to a belief of 40 days of latency for households using containers for consumption only. Yet, we do not find strong statistical evidence for this difference (*P* = 0.188).

Table 2 shows descriptive statistics for the main covariates at the household level. We record a large proportion of maize sellers during the post-harvest season, which includes the early post-harvest period and the lean period. Maize sellers amount to 432 households (70% of the sample).^17^ The decision of whether to sell, and the amount to sell remain almost unchanged over the period of the data collection, as there is no statistically significant difference between the two waves of data collection in 2011 and 2013. More specifically, data show that 83% of households who sold maize in year one sold again in year two. Similarly, 67% of households who did not sell maize in the first wave, continued to withhold maize from the market in the second wave. The real average expenditure on chemical insecticide (2011 being the base year), the main variable for chemical use, is about 1,270 F CFA (US$ 2.20). Data also indicate that the amount spent on chemical insecticide and its use did not changed between the two waves of data collection. The pattern of market participation is therefore consistent with the storage practices not changing much over time. This supports our claim that households’ perception of the risk of food contamination from chemical use has not changed during the 2 years between rounds of our survey.

## 7 Results

### 7.1 Main results

Table 3 presents the results of the testing of Hypotheses 1 and 2, about the factors that affect a household’s decision to apply chemical insecticide to a maize container. Results are from MC-probit models and estimates represent average partial effects (APE). Columns (1) to (2) are, respectively, the parsimonious and the full regression results that test Hypothesis 1. Columns (3) and (4) account for the interaction term to test Hypothesis 2. Results in column (1) show that a household’s likelihood of applying chemical insecticide to a maize container intended for sales is about 37 percentage points higher on average than it is when the container is intended for consumption only. This estimate is statistically significant with a *P*-value < 0.01. This result remains almost unchanged when we control for more covariates in column (2). Therefore, we reject Hypothesis 1 that a household’s goal for their maize has no statistically significant effect on its decision to apply insecticides to containers intended for sale compared to containers intended for home consumption. In other words, maize that is stored to be sold later is more likely to be sprayed with chemical insecticide than maize held in a container kept for home-consumption.

**Table 2:** Descriptive statistics at the household level

Variables	Pooled sample			2011		2013		P-value^a^
	Mean	Median	SD	Mean	SD	Mean	SD	
Quantity sold during post-harvest (kg)	1,610	400	3,703	1,504	3,879	1,717	3,522	0.17
=1 if HH sells maize	0.70			0.70		0.70		0.83
*Tested covariates*								
Expenditures on insecticides (x 1,000 F CFA)	1.27	0	5.67	1.1	3.9	1.5	7.0	0.32
Number of days for grain safety (latency periods)	36	31	30	36	31	36	31	n/a
=1 if HH uses chemical insecticide	0.25			0.24		0.26		0.28
=1 if HH does not consume chemic. sprayed maize	0.44			0.44		0.44		n/a
*Container-level variables*								
=1 if HH’s storage goal is consumption only	0.33			0.34		0.32		0.57
=1 if HH’s storage goal is sale only	0.04			0.04		0.04		0.66
=1 if HH’s storage goal is sale & consumption	0.63			0.62		0.64		0.49
=1 if HH uses more than 1 container	0.14			0.21**		0.07**		0.00
=1 if HH bought certified chemical	0.04			0.06*		0.03*		0.09
Quantity stored at the end ofharvest (kg)	2,555	1107.5	4,430	2,343**	4,505	2,768**	4,350	0.01
*Information system*								
=1 if HH reports a case of chemical intoxication	0.02			0.02		0.02		n/a
HH has received informa. about chemic. (no. of years)	0.63	0	2.30	0.50**		0.77**		0.00
Education level (no. of years)	1.57	0	2.27	1.57	2.27	1.57	2.27	n/a
Number of children in school	4	3	3	3_**_	3	4_**_	3	0.00
HH owns a radio (no. of years)	5.65	2	7.78	494**	7.54	6.36**	7.96	0.00
HH owns a TV (no. of years)	1.45	0	3.43	1 19**	3.09	1.71_**_	3.73	0.00
=1 if HH owns a cell phone	0.56			0.38**		0.75**		0.00
=1 if input dealer is in village	0.13			0.17**		0.09**		0.00
=1 if extension agent is in village	0.42			0.58**		0.26**		0.00
*Liquidity and other physical assets*								
Farm size (ha)	4.43	3	5.21	3.7_**_	4.0	5.2**	6.1	0.00
Savings at the start of harvest (x 1,000 F CFA)	132	24.09	298	88	24	176	335.6	0.00
*Market variables*								
Post-harvest price (FCFA/kg)	143.63	137.88	51.86	136.4**	53.5	150.9**	49.2	0.00
Distance from the main market (km)	5.87	6.24	5.00	5.9	5.0	5.9	5.0	n/a
*Households’ demographics*								
Household size	11	9	6	11	6	11	6	0.36
Age of the household head	43.70	42	13.39	42.7	13.4	44.7	13.4	n/a
=1 if household head is male	0.91			0.91		0.91		n/a

***Notes:*** *^a^P-value* for paired t-test for the difference between panel year: **P < 0.01, *P < 0.1; McNemar’s Chi-squared provides similar *P*-values for the discrete covariates; n/a stands for not applicable; 1 US$ = 512 F CFA at the time of the survey; only frequency is provided.

**Table 3:** Factors that affect a household’s decision to apply insecticide to a maize container

Dep = 1 if content of container is sprayed with chemical	(1)	(2)	(3)	(4)
=1 if container intended for sale only	0.37***	0.35***	0.19*	0.23**
	(0.08)	(0.07)	(0.12)	(0.1)
=1 if container intended for sale & consumption	−0.1*	−0.09*	−0.10	−0.08
	(0.06)	(0.05)	(0.07)	(0.06)
Number of latency days needed for insecticides to be safe	2.5E–04	5E–05	−5.2E–04	7.6E–05
	(1E–03)	(1E–03)	(1E–03)	(1E–03)
=container for sale x (no. of days for grain safety)			4E–03**	4E–03*
			(2E–03)	(2E–03)
=container for sale & consumption x (no. days for grain safety)			1.4E–04	−2.7E–04
			(1E–03)	(1E–03)
=1 if HH reports a case of chemical intoxication in village		−0.17		−0.17
		(0.11)		(0.11)
HH has received information about chemical (no. of years)		−0.02		−0.02
		(0.02)		(0.02)
HH owns a radio (no. of years)		9E–03		9E–03
		(0.02)		(0.02)
HH owns a TV (no. of years)		−6E–03		−9E–03
		(0.02)		(0.02)
=1 if HH owns a cell phone		−0.02		−0.02
		(0.04)		(0.04)
Education level (no. of years)		−7E–03		−7E–03
		(9E–03)		(9E–03)
Number of children in school		−0.03*		−0.03*
		(0.02)		(0.02)
=1 if input dealer is in village		0.35**		0.34***
		(0.14)		(0.13)
=1 if extension agent is in village		0.01		0.01
		(0.04)		(0.04)
=1 if household bought certified chemical		0.18**		0.18**
		(0.08)		(0.08)
Savings at the start of harvest season (x 1,000 F CFA)		−2.3E–06		−3.1E–06
		(−2E–05)		(−2E–05)
Farm size (ha)		−4E–03		−4E–03
		(3E–02)		(3E–02)
Distance from the main market (km)		−2E–03		−2E–03
		(5E–03)		(5E–03)
Age of household head		−1E–03		−1E–03
		(1E–03)		(1E–03)
=1 if household head is male		0.15**		0.15**
		(0.06)		(0.06)
Household size		2E–03		2E–03
		(4E–03)		(4E–03)
=1 if container type is traditional granary		0.09*		0.09*
		(0.05)		(0.05)
=1 if container type is polypropylene plastic bag		0.05		0.05
		(0.04)		(0.04)
=1 if container type is ceiling		0.07		0.07
		(0.05)		(0.05)
=1 if HH uses more than one container		−0.04		−0.05
		(0.04)		(0.04)
Observations	707	707	707	707
Pseudo R^2^	0.16	0.35	0.17	0.35

***Notes:*** Results are obtained from probit combined with Mundlack-Chamberlin (MC) device. All predictors are Average Partial Effect (APE), standard errors in parentheses; estimates are obtained using the Stata margin and lincom commands, and for the interaction terms they are similar to those obtained with the command inteff (Norton *et al.,* 2004); ***P < 0.01, **P < 0.05, *P < 0.1; village dummies, average and time effects are included; US$ 1.00 = 512 F CFA at the time of the survey.

We obtain additional insights when households are differentiated by their perception of the risk of food safety. Hypothesis 2 tests how a household’s awareness of the risk of food contamination influences its decision to apply chemical insecticides to maize containers allocated to future sales. Results in columns (3) and (4) indicate that the probability that a household applies chemical insecticide to a maize container intended for sales increases with its awareness of the health risk of chemical insecticides. In column (3), we find that if there is no perception of a risk of food contamination, the likelihood that a household uses insecticide on maize held in a container intended for sales is about 20 percentage points higher on average than it is for maize held in a container intended for consumption. Households might still apply chemical insecticides to maize they intend to sell later during the post-harvest season irrespective of any food safety concern, expecting to have grain to sell later in the season. However, further analyses of the results in column (3) reveal that food safety concerns might also influence households’ use of insecticides on maize. In fact, the coefficient estimate of a household’s likelihood of using chemical insecticide on maize that it intends to sell increases by about 0.4 percentage points per day of latency when there is a perceived risk of food contamination. The interaction term between a household’s storage goal being to sell and its subjective belief about the risk of food contamination is, indeed, positive and statistically significant, with *P*-value = 0.05. More specifically, the joint estimate of this interaction term and the covariate for storage goal being to sell is statistically significant with a *P*-value < 0.01.

When we evaluate the coefficient estimates at the mean of the variable ‘number of latency days a container needs after applying insecticides to be safe to eat’ (36 days), we find that a household with this belief is on average 33 percentage points more likely to use insecticide spray on maize stored for sale rather than for consumption. In other words, the more a household suspects the health risk from applying insecticides to maize, the more likely it is to spray maize in a container intended for sales than it is to spray maize in a container intended for consumption. As shown in the conceptual framework, once the probability of the household believing that the health risks of insecticides are greater than zero, the valuation of maize quality in the household could shift further away from home consumption to markets. Our results in column (4) are not substantially different in magnitude and statistical significance from the parsimonious results presented in column (3). Clearly, we reject Hypothesis 2, since the probability of a household applying insecticides to maize that it intends to sell increases with its subjective belief about the food safety risk associated with using insecticides (see also online Appendix D for graphical results).

Table 4 presents the results of the testing of Hypotheses 3 and 4. This table shows how a household’s investments in chemical insecticides and its subjective belief about food safety affect the quantity of maize that is sold during the post-harvest season. Results in columns (1) and (2) test Hypothesis 3. More specifically, column (1) corresponds to the parsimonious regression that includes the tested covariates along with key variables of the container-level behaviour tested under the previous hypotheses. Column (2) represents the full regression with additional controls. Results in columns (3) and (4) include an interaction term between expenditures on insecticides and a household’s subjective belief about the risk of food contamination and therefore test Hypothesis 4. In columns (1) and (2), we find that expenditure on chemical insecticides has a positive and statistical significant effect on the quantity of maize sold into markets once the household decides to sell its stocks. In the parsimonious results in column (1), we find that an additional 1,000 F CFA (US$ 2.00) spent on insecticides increases the quantity of maize that is sold by about 5% on average. This is consistent with our first set of findings indicating that a household is more likely to apply chemical insecticide to maize that it intends to sell, rather than maize it intends to consume. Therefore, we reject Hypothesis 3 that the covariate, expenditures on chemical insecticide, has no statistically significant effect on the quantity of maize sold into markets.

**Table 4:** Factors that affect the amount of maize sold during the post-harvest season

Dependent variable = Log (Quantity sold)	(1)	(2)	(3)	(4)
Expenditures on insecticides (x 1000 FCFA)	0.05***	0.06***	0.03**	0.04***
	(0.02)	(0.01)	(0.01)	(0.01)
Number of latency days needed for insecticides to be safe	3.2E–04	2E–03	−1E–03	1E–03
	(2E–03)	(2E–03)	(2E–03)	(2E–03)
Expenditures on insecticides x (no. of days for grain safety)			1E–03***	5E–04**
			(2E–04)	(2.2E–04)
=1 if HH stores for sale	0.30	0.43	0.27	0.39
	(0.74)	(0.66)	(0.73)	(0.65)
=1 if HH stores for consumption & sale	−0.26	−0.18	−0.29	−0.21
	(0.42)	(0.39)	(0.42)	(0.4)
=1 if HH uses more than one storage container	0.36*	0.47**	0.36*	0.46**
	(0.21)	(0.18)	(0.21)	(0.18)
Quantity of maize stored (kg)	2.1E–04***	2.1E–04***	2.2E–04***	2.2E–04***
	(3E–05)	(3E–05)	(3.4E–05)	(3.2E–05)
=1 if HH bought certified chemical	−0.57*	0.79***	−0.62**	−0.83***
	(0.29)	(0.26)	(0.3)	(0.26)
=1 if HH reports a case of chemical intoxication in village		0.70		0.68
		(0.50)		(0.51)
HH has received information about chemical (no. of years)		−0.23*		−0.22*
		(0.13)		(0.13)
HH owns a radio (no. of years)		−0.27**		−0.28**
		(0.13)		(0.13)
HH owns a TV (no. of years)		−0.05		−0.02
		(0.11)		(0.11)
=1 if HH owns a cell phone		0.37*		0.36*
		(0.21)		(0.21)
Education level (no. of years)		−0.04		−0.03
		(0.02)		(0.02)
Number of children in school		0.11		0.10
		(0.08)		(0.08)
=1 if input dealer is in village		−0.23		−0.24
		(0.24)		(0.24)
=1 if extension agent is in village		−0.04		−0.03
		(0.15)		(0.15)
Post-harvest price (F CFA/kg)		3E–03*		3E–03*
		(2E–03)		(2E–03)
Distance from the main market (km)		4E–03		2E–03
		(0.02)		(0.02)
Savings at the start of harvest season (x 1,000 F CFA)		2.4E–04		3E–04
		(4E–04)		(4E–04)
Farm size (ha)		0.02**		0.02**
		(0.01)		(0.01)
Age of household head		−1E–03		−1E–03
		(5E–03)		(5E–03)
=1 if household head is male		0.51***		0.50***
		(0.18)		(0.18)
Household size		−4E–03		−5E–03
		(0.02)		(0.02)
Constant	5.69***	4.49***	5.73***	4.56***
	(0.23)	(0.57)	(0.24)	(0.58)
Observations	432	432	432	432
Adjusted R^2^	0.71	0.73	0.71	0.73

***Notes:*** Results are obtained from OLS-MC; standard errors in parentheses; ***P < 0.01, **P < 0.05, *P < 0.1; village dummies, time and average effects are included; US$ 1.00 = 512 F CFA at the time of the survey.

Next, we investigate how a household’s subjective belief about the risk of food contamination affects their market transactions when they invest in chemical insecticides. The corresponding results are presented in columns (3) and (4) of Table 4. In column (3), we find that a household that is unaware of the health risks of insecticides (e.g. believes that the number of latency days required for chemicals to be safe is zero) and yet decides to use them, sells significantly more maize than unaware households who do not use them. For example, a 1,000 F CFA (US$ 2.00) increase in expenditures on insecticides by unaware households increases the average quantity of maize that they sell by about 3%, *ceteris paribus*. It is reasonable to believe that these households want to recover some part of their investment in insecticides irrespective of any food safety concern. Nevertheless, further analysis suggests that the optimum quantity to sell into markets changes for households who do not perceive a risk of food contamination. Results in column (3) indicate that the interaction term between expenditures on chemical insecticides and the household’s food safety concern (number of days of latency needed) is positive and statistically significant. Moreover, the joint estimate of expenditures on chemical insecticide and its interaction term with food safety is statistically significant with a *P*-value < 0.01. This implies that maize sales increase by 0.1% for every additional day that households believe that insecticides should remain latent and for every 1,000 F CFA (US$ 2.00) spent on chemical insecticides. These results show that households who suspect a food safety risk from applying insecticides to maize will sell more of their maize stocks than those who do not. We argue, based on the conceptual framework, that a household’s subjective belief about the risk of food contamination reduces its marginal cost of investments in quality improvement, inducing it to sell more maize to the market. Hence, we reject Hypothesis 4: that ‘bad sellers’ defined as maize sellers who are aware of food safety risks from consuming maize treated with insecticides sell as much maize into markets as households who are unaware of this food safety risk and are labelled as ‘uninformed sellers’. Clearly, these ‘bad sellers’ appear to sell more maize into the market.

### 7.1 Robustness checks

Our findings remain unchanged under different sets of robustness checks. The first compares the parsimonious regressions to the full regressions. We present the corresponding results in the previous tables.^18^ An additional robustness check is to replace the continuous variable of food safety risk by the binary variable that also measures a household’s attitude with respect to consuming maize that has been sprayed with chemical insecticide.^19^ Using this discrete variable, we still obtain the same findings for Hypotheses 1 and 2 at the container level (see online Appendix F1). Likewise, our results for Hypotheses 3 and 4 are consistent with the previous findings for actual market transactions measured at the household level (see online Appendix F2).

## 8 Conclusion

Adverse selection, where sellers of maize apply unobservable chemical insecticides to maize they intend to sell but not to maize they intend to consume, is a potentially important and under-recognised problem in informal food markets in developing countries. In this context there is a lack of enforceable quality standards, third-party verification, and insufficient price premiums to incentivise sellers to invest in practices that improve quality and reduce risks to consumers. We develop a simple conceptual model of maize marketing and use household-level panel data from Benin to estimate how adverse selection could occur in an informal market where participants observe different features of quality attributes. Asymmetric information arises from sellers’ decisions to adopt practices that could alter unobservable maize quality, such as applying chemical insecticides to maize, which protect maize from insect attacks, but may have adverse health risks when consumed by humans. We also consider that sellers are likely to have imperfect information about the health risks associated with applying chemical insecticides to maize. We empirically estimate how a seller’s subjective belief about the risk of food safety from applying chemical insecticides to maize could lead to adverse selection in the market.

We first rely on a conceptual framework to show how a seller’s subjective belief about the risk of food contamination affects maize allocation in the household. Moreover, empirical evidence reveals adverse selection in Benin’s maize markets that we trace from storage practices to market transactions. We find that households who are aware of potential contamination from chemical insecticides are more likely to apply insecticides to maize intended for sale than on home-consumed maize. Consequently, such households sell a larger amount of their stock into markets when they use insecticides than households who are unaware of or unconcerned about pesticide contamination. It is indeed rational for a household to sell more maize sprayed with chemical insecticides into the market to preserve the quantity and quality, knowing that the grain will look good to buyers, and that buyers will be unable to fully detect if chemical residues are present.

Hence, adverse selection in informal maize markets may be driven in part by households’ subjective beliefs about the risk of food contamination. Our results are consistent with a previous work by Hoffmann and Gatobu (2014) and help explain why many rural sellers value homegrown grain compared to market purchased maize. Clearly, the prevalence of low quality maize in markets added to the insufficient premium for higher quality further reduce incentives for households’ market participation.

Overall, we emphasise that sellers’ subjective beliefs about unobservable quality attributes might create more than adverse selection in informal markets. Adverse selection impedes market participation and may result in serious health risks for consumers. Nevertheless, information about food safety and appropriate storage practices remain largely imperfect in informal markets to the extent that smallholder sellers are also exposed to risks from consuming unsafe grains from markets or home stocks. This situation is partly due to the limited supply of improved post-harvest inputs such as moisture meters and improved storage containers compared with production inputs, like inorganic fertiliser and improved seed, both of which are widely promoted across SSA. Since increased food production is of limited value if it is unsafe to eat, policy-makers, donors and development practitioners need to shift some emphasis and resources into increasing food quality rather than just increasing food quantity.

## Footnotes

2 Tanguy *et al*. (2017) use evidence from the onion markets in Senegal to show that farmers who knew in advance that their products would be graded by an external entity invested more in quality-enhancing technology.

3 In a study of 2,689 farmers in Nigeria, only 32% reported knowledge of mycotoxins (Idahor and Ogara, 2010). In Benin, Ghana and Togo, awareness rates were 20.8% among a sample of farmers and 26.7% among traders (James *et al*., 2007).

4 One might view the fact that a household aware of the risk of food contamination applies chemical insecticide on a maize container as a moral hazard behaviour. However, in this context we emphasise the adverse selection side of the story in the sense that a household’s decision to use chemical insecticide may lead to bad quality being sold into markets.

5 Our data suggest that storage losses are about 7% on average (with 8.5% as the standard deviation), and that 10% of the sampled farmers face storage losses higher than 15%.

6 We simplify attributes (*a*) and (*b*) to only unobservable attributes, the main interest in this article.

7 For example, some practices such as inappropriate drying or insecticide use could affect maize food safety and cause health risk. Inappropriate drying here refers to practices such as drying grain directly on the bare ground or not allowing sufficient time for drying. Drying on the bare ground increases the risk of contamination by fungi that produce aflatoxin, whereas insufficient drying results in wet maize that is more susceptible to mould.

8 The fact that many households (40% in our sample) buy back maize shows that they do not necessarily secure consumption first.

9 We assume that *u^E^* ≡ *u* in the post-harvest season, i.e. that the household achieves the expected quality.

10 A household may obtain additional maize because insecticides reduce pest damage and therefore reduce quantity losses. Then, the household cannot consume a quantity of maize that exceeds its needs during the post-harvest season nor can it keep maize over an extended period. Hence, the effect on sales is more likely to be positive.

11 Guidelines suggest maize treated with Actellic Gold Dust can be consumed 2 weeks following application.

12 A test of the joint significance of *β*_1_ and *β*_3_ is a sufficient but not necessary condition to determine the presence of an interaction effect in a non-linear model (Greene, 2010). Like the marginal effect of a single variable, the magnitude of the interaction effect depends on all the covariates in the model. But it can have different signs for different observations, making summary measures of the interaction effect difficult (Ai and Norton, 2003). Therefore, unlike single variables, conditional effects cannot be simply inferred by assessing the sign and statistical significance of the coefficient of the interaction term (Ai and Norton, 2003; Greene, 2010). Building on Ai and Norton (2003), Greene (2010) indicates that graphical presentations of the predictive effects are a very informative adjunct to the numerical results of the interaction effect.

13 The quality produced by the two types of sellers is not necessarily the same even though they may adopt the same practices. Because the quality attribute is unobservable and many households are uninformed, what matters is the quality as perceived by theeller.

14 We also account for correlation among containers belonging to the same household (repeated observations) by clustering the estimation at the household level.

15 We also follow a control function approach and test the endogeneity of chemical expenditure. We use the number of years the household head has belonged to an association or a group in the village as the instrumental variable. This instrument measures how easily a household may have access to insecticide through community networks. However, we do not believe that the instrument could have a direct effect on households’ sale transactions, since for most households these associations have no direct link with maize marketing. We do not find that chemical expenditure is endogenous (see online Appendix A).

16 We drop one household that reported cultivated area of more than 51 times the sample average.

17 About 70% of most important (in volume) sales were made to local or itinerant traders.

18 For Hypotheses 1 and 2 the findings also remain consistent under a linear probability model (see online Appendix E).

19 Using a binary variable for a household’s subjective belief allows less variability than a continuous measure would and might therefore introduce greater bias if the variable is not exogenous. Bontemps and Nauges (2015) suggest a special regressor approach to deal with how a potential endogenous binary variable used for perception might affect a risk-averting decision which is also a binary variable. Here, we were unable to find an appropriate special regressor and an instrument to follow their approach.

## Supporting Information

**Appendix A.** Factors that affect the amount of maize sold – control function approach.

**Appendix B.** Factors that affect the decision to sell into markets during the post-harvest season (Probit-MC).

**Appendix C.** Probability of a household being re-interviewed (Probit-MC).

**Appendix D.** Predicted probability of insecticide application to a maize container. **Appendix E.** Factors that affect a household’s decision to apply insecticide to a container (OLS-MC).

**Appendix F1.** Factors that affect a household’s decision to apply insecticide to a maize container (Probit-MC).

**Appendix F2.** Factors that affect the quantity of maize sold in post-harvest season (OLS-MC).
