# Supplementary material for: Food Safety and Adverse Selection in Rural Maize Markets
Source: J Agric Econ. 2019 Jul 29:1–27. doi: 10.1111/1477-9552.12350 (PMC7198124; doi:10.1111/1477-9552.12350)
Supplement: Supplementary file 1 [file JAE-2019-1477-9552-12350-s1.docx]

**Food Safety and Adverse Selection in Rural Maize Markets**

Didier Kadjo, Jacob Ricker-Gilbert, Gerald Shively and Tahirou Abdoulaye

**Online Appendix**

**Appendix A.** Factors that affect the amount of maize sold – control function approach

|  | **Dep. Var.=Expenditure**  **on insecticides** | **Dep. Var.=**  **Log(Qty sold)** |
| --- | --- | --- |
| Number of years of membership in an association | 0.034** |  |
|  | (0.017) |  |
| Residual |  | 0.021 |
|  |  | (1.049) |
| Expenditures on insecticides (x 1,000 F CFA) |  | 0.058*** |
|  |  | (0.017) |
| No. of latency days needed for insecticides to be safe | -4E-04 | 0.002 |
|  | (0.004) | (0.036) |
| =1 if HH stores for sale only | 0.498 | 0.521 |
|  | (2.458) | (27.239) |
| =1 if HH stores for sale & consumption | 0.206 | -0.146 |
|  | (0.439) | (3.467) |
| =1 HH uses more than 1 container | 0.370 | 0.505 |
|  | (0.373) | (1.935) |
| Quantity of maize stored (Kg) | -1E-04 | 2E-05 |
|  | (1E-04) | (5E-05) |
| =1 if HH bought certified chemical | 1.336** | -0.650 |
|  | (0.528) | (6.090) |
| =1 if HH reports a case of chemical intoxication in village | 0.693 | 0.763 |
|  | (0.459) | (2.209) |
| HH has received information about chemical (# Years) | 0.276 | -0.204 |
|  | (0.212) | (1.272) |
| HH owns a radio (# Years) | 0.380 | -0.230 |
|  | (0.245) | (3.8420) |
| HH owns a TV (# Years) | -0.113 | -0.062 |
|  | (0.215) | (1.580) |
| =1 if HH owns a cell phone | -0.055 | 0.363 |
|  | (0.402) | (2.126) |
| Education level (# Years) | -0.063 | -0.0460 |
|  | (0.044) | (0.594) |
| Number of children in school | -0.416** | 0.062 |
|  | (0.164) | (1.60099) |
| =1 if input dealer is in village | 1.927*** | -0.018 |
|  | (0.628) | (12.048) |
| =1 if extension agent is in village | 0.413 | 0.008 |
|  | (0.335) | (0.624) |
| Post-harvest price (F CFA/Kg) | 0.004 | 0.003 |
|  | (0.003) | (0.020) |
| Distance from the main market (Km) | 0.003 | 0.004 |
|  | (0.034) | (0.105) |
| Savings at the start of harvest season (x 1,000 F CFA) | -4E-04 | 2E-04 |
|  | (0.001) | (0.006) |
| Farm size (Ha) | -0.035* | 0.022 |
|  | (0.020) | (0.058) |
| =1 if panel is second wave | 0.022 | 0.474 |
|  | (0.477) | (3.981) |
| Observations | 618 | 618 |
| Pseudo R2/R2 | 0.168 | 0.760 |

***Notes***: Column (1) is the reduced form obtained from a Tobit model combined with a MC device. Column (2) is structural model obtained from an OLS-MC. Standard errors in parentheses; *** *p*<0.01, ** *p*<0.05, * *p*<0.1; all predictors are average partial effect; village dummies, average effects and time effects are included; households’ demographics also included; 1 US$ = 512 F CFA at the time of the survey.

**Appendix B.** Factors that affect the decision to sell into markets during the post-harvest season (Probit-MC)

| **Dependent variable= 1 if HH sold maize** | **(1)** | **(2)** | **(3)** | **(4)** |
| --- | --- | --- | --- | --- |
| Expenditures on insecticides (x 1000 FCFA) | 0.008 | 0.010 | 0.010 | 0.013 |
|  | (0.008) | (0.009) | (0.010) | (0.011) |
| Number of latency days needed for insect. to be safe | -0.001*** | -4E-04 | -0.001** | -3.5E-04 |
|  | (3E-04) | (3E-04) | (3E-04) | (4E-04) |
| Expenditures on insecticides x (# days for grain safe) |  |  | -4 E-05 | -7E-05 |
|  |  |  | (1E-04) | (1.2 E-04) |
| =1 if HH stores for sale | 0.567*** | 0.584*** | 0.566*** | 0.584*** |
|  | (0.085) | (0.117) | (0.084) | (0.117) |
| =1 if HH stores for consumption & sale | 0.554*** | 0.523*** | 0.553*** | 0.522*** |
|  | (0.081) | (0.073) | (0.081) | (0.072) |
| =1 if HH uses more than one storage container | 0.103 | 0.097 | 0.103 | 0.096 |
|  | (0.064) | (0.065) | (0.064) | (0.065) |
| Quantity of maize stored (Kg) | 3E-05** | 4E-05*** | 3E-05** | 4E-05*** |
|  | (1.1E-05) | (1.2E-05) | (1.1E-05) | (1.2E-05) |
| =1 if HH bought certified chemical | -0.080 | -0.096 | -0.079 | -0.095 |
|  | (0.089) | (0.068) | (0.089) | (0.068) |
| =1 if HH reports a case of chemical intoxic. in village |  | -0.055 |  | -0.056 |
|  |  | (0.042) |  | (0.042) |
| HH has received inform. about chemical (# Years) |  | -0.003 |  | -0.004 |
|  |  | (0.032) |  | (0.033) |
| HH owns a radio (# Years) |  | -0.006 |  | -0.006 |
|  |  | (0.028) |  | (0.028) |
| HH owns a TV (# Years) |  | -0.013 |  | -0.013 |
|  |  | (0.026) |  | (0.026) |
| =1 if HH owns a cell phone |  | 0.004 |  | 0.003 |
|  |  | (0.048) |  | (0.048) |
| Education level (# Years) |  | -0.001 |  | -0.001 |
|  |  | (0.005) |  | (0.005) |
| Number of children in school |  | 0.002 |  | 0.002 |
|  |  | (0.014) |  | (0.014) |
| =1 if input dealer is in village |  | 0.077 |  | 0.077 |
|  |  | (0.065) |  | (0.065) |
| =1 if extension agent is in village |  | -0.008 |  | -0.008 |
|  |  | (0.045) |  | (0.045) |
| Post-harvest price (F CFA/Kg) |  | 0.001*** |  | 0.001*** |
|  |  | (0.000) |  | (0.000) |
| Distance from the main market (Km) |  | 0.005* |  | 0.005* |
|  |  | (0.003) |  | (0.003) |
| Savings at the start of harvest season (x 1,000 F CFA) |  | 1E-04*** |  | -1E-04 |
|  |  | (6E-05) |  | (6E-05) |
| Farm size (Ha) |  | 0.005 |  | 0.005 |
|  |  | (0.005) |  | (0.005) |
| Age of household's head |  | -4E-04 |  | -4E-04 |
|  |  | (0.001) |  | (0.001) |
| =1 if Household’s head is male |  | -0.001 |  | -0.002 |
|  |  | (0.037) |  | (0.037) |
| Household size |  | -0.011** |  | -0.011** |
|  |  | (0.005) |  | (0.005) |
| Observations | 618 | 618 | 618 | 618 |
| Pseudo R2 | 0.163 | 0.661 | 0.614 | 0.661 |

***Notes***: Results are obtained from probit combined with Mundlack-Chamberlin (MC) device; all predictors are Average Partial Effect (APE), standard errors in parentheses; estimates are obtained using the Stata margin commands, and for the interaction term they are similar to those obtained with the command inteff (Norton *et al*., 2004); *** *p*<0.01, ** *p*<0.05, * *p*<0.1; village dummies, average, and time effects are included; US$ 1.00 = 512 F CFA at the time of the survey.

**Appendix C.** Probability of a household being re-interviewed (Probit-MC)

| **Dependent variable = 1 if HH is re-interviewed** | **Average Partial effect** |
| --- | --- |
| =1 if HH stores for sale | -0.011 |
|  | (0.110) |
| =1 if HH stores for consumption & sale | -0.010 |
|  | (0.046) |
| Expenditures on insecticides (x 1,000 F CFA) | 0.006 |
|  | (0.009) |
| =1 if HH bought certified chemical | -0.076 |
|  | (0.070) |
| =1 if input dealer is in village | 0.020 |
|  | (0.085) |
| =1 if extension agent is in village | -0.017 |
|  | (0.054) |
| Post-harvest price (F CFA/Kg) | -2.5E-04 |
|  | (4.5 E-04) |
| Distance from the main market (Km) | 0.003 |
|  | (0.005) |
| Quantity of maize stored (Kg) | -5.1E-06 |
|  | (5.8 E-06) |
| Savings at the start of harvest season (x 1,000 F CFA) | 4 E-04* |
|  | (2 E-04) |
| Farm size (Ha) | 0.015 |
|  | (0.010) |
| Age of household's head | 0.003* |
|  | (0.001) |
| Education level (# Years) | -0.002 |
|  | (0.009) |
| =1 if household’s head is male | 0.026 |
|  | (0.063) |
| Household size | -0.002 |
|  | (0.003) |
| =1 if department is Atlantique | 0.110 |
|  | (0.071) |
| =1 if department is Mono | 0.196** |
|  | (0.087) |
| =1 if department is Colline | 0.122 |
|  | (0.085) |
| =1 if department is Atacora | 0.074 |
|  | (0.064) |
| =1 if department is Borgou | 0.128 |
|  | (0.078) |
| Observations | 354 |
| Pseudo R2 | 0.413 |

***Notes***: Estimated are Average Partial Effect from probit; standard errors in parentheses*** *p*<0.01, ** *p*<0.05, * *p*<0.1; 1 household was an outlier for production size, 5 observations were dropped because of missing or inconsistent information during the second wave of data collection; some covariates were recall variables during the second wave, and therefore could not be collected for dropped households; US$ 1.00 = 512 F CFA at the time of the survey.

**Appendix D.** Predicted probability of insecticide application to a maize container

***Note***: The interaction effect shows up as the increasing gap between the predicted probabilities for a container with content intended for sale and a container kept for home consumption.

**Appendix E.** Factors that affect a household’s decision to apply insecticide to a container (OLS-MC)

| **Dep. =1 if content of container is sprayed with chemical** | **(1)** | **(2)** | **(3)** | **(4)** |
| --- | --- | --- | --- | --- |
| =1 if container intended for sale only^a^ | 0.498*** | 0.477*** | 0.299** | 0.372*** |
|  | (0.092) | (0.089) | (0.136) | (0.125) |
| =1 if container intended for sale & consumption | -0.089 | -0.097* | -0.100 | -0.099 |
|  | (0.062) | (0.057) | (0.075) | (0.071) |
| Number of latency days needed for insecticides to be safe | 2E-04 | 3E-04 | -3E-04 | 4E-05 |
|  | (0.001) | (0.001) | (0.001) | (0.001) |
| =container for sale x (# days for grain safety)^a^ |  |  | 0.004** | 0.002 |
|  |  |  | (0.002) | (0.002) |
| =container for sale & consumptio x (# days for grain safe) |  |  | 3E-04 | 1E-04 |
|  |  |  | (0.001) | (0.001) |
| =1 if HH reports a case of chemical intoxication in village |  | -0.167 |  | -0.167 |
|  |  | (0.134) |  | (0.135) |
| HH has received information about chemical (# Years) |  | -0.023 |  | -0.023 |
|  |  | (0.028) |  | (0.027) |
| HH owns a radio (# Years) |  | 0.027 |  | 0.030 |
|  |  | (0.023) |  | (0.024) |
| HH owns a TV (# Years) |  | -0.010 |  | -0.013 |
|  |  | (0.021) |  | (0.021) |
| =1 if HH owns a cell phone |  | -0.035 |  | -0.033 |
|  |  | (0.043) |  | (0.044) |
| Education level (# Years) |  | -0.007 |  | -0.007 |
|  |  | (0.010) |  | (0.010) |
| Number of children in school |  | -0.029 |  | -0.029 |
|  |  | (0.019) |  | (0.019) |
| =1 if input dealer is in village |  | 0.190*** |  | 0.191*** |
|  |  | (0.072) |  | (0.072) |
| =1 if extension agent is in village |  | 0.017 |  | 0.017 |
|  |  | (0.034) |  | (0.034) |
| =1 if HH bought certified chemical |  | 0.196* |  | 0.194* |
|  |  | (0.105) |  | (0.105) |
| Distance from the main market (Km) |  | -0.003 |  | -0.003 |
|  |  | (0.004) |  | (0.004) |
| Savings at the start of harvest season (x 1,000 F CFA) |  | -1E-05 |  | -1E-05 |
|  |  | (2E-05) |  | (2E-05) |
| Farm size (Ha) |  | -0.006** |  | -0.005** |
|  |  | (0.003) |  | (0.003) |
| Age of household's head |  | -0.001 |  | -0.001 |
|  |  | (0.002) |  | (0.002) |
| =1 if household’s head is male |  | 0.123* |  | 0.125* |
|  |  | (0.063) |  | (0.064) |
| Household size |  | 0.002 |  | 0.001 |
|  |  | (0.004) |  | (0.004) |
| Constant | -0.058 | 0.0460 | -0.0559 | 0.0367 |
|  | (0.136) | (0.1985) | (0.1354) | (0.2003) |
| Dummies for type of containers included | Yes | Yes | Yes | Yes |
| Village dummies included | Yes | Yes | Yes | Yes |
| Observations | 707 | 707 | 707 | 707 |
| Adjusted R-squared | 0.171 | 0.327 | 0.174 | 0.327 |

***Notes***: Results are obtained from OLS-MC, *** *p*<0.01, ** *p*<0.05, * *p*<0.1; ^a^*p*-value for the interaction term is equal to 0.16 and *p*-value on the F-joint test is less than 0.01; average and time effects are included.

**Appendix F1.** Factors that affect a household’s decision to apply insecticide to a maize container (Probit-MC)

| **Dep = 1 if content of container is sprayed with chemical** | **(1)** | **(2)** | **(3)** | **(4)** |
| --- | --- | --- | --- | --- |
| =1 if container intended for sale only^a^ | 0.376*** | 0.350*** | 0.342*** | 0.332*** |
|  | (0.077) | (0.074) | (0.107) | (0.098) |
| =1 if container intended for sale & consumption | -0.096* | -0.094** | -0.080 | -0.095 |
|  | (0.058) | (0.048) | (0.071) | (0.058) |
| =1 if HH perceives risk of food safety from chemical | -0.007 | 0.029 | 0.010 | 0.026 |
|  | (0.040) | (0.033) | (0.060) | (0.048) |
| Container for sale x (=1 if HH perceives risk of food safety)^a^ |  |  | 0.0654 | 0.048 |
|  |  |  | (0.148) | (0.128) |
| Container for sale & consumption x (=1 if HH perceives risk) |  |  | -0.036 | -0.006 |
|  |  |  | (0.077) | (0.065) |
| =1 if HH reports a case of chemical intoxication in village |  | -0.159 |  | -0.160 |
|  |  | (0.112) |  | (0.111) |
| HH has received information about chemical (# Years) |  | -0.021 |  | -0.021 |
|  |  | (0.024) |  | (0.024) |
| HH owns a radio (# Years) |  | 0.009 |  | 0.010 |
|  |  | (0.022) |  | (0.022) |
| HH owns a TV (# Years) |  | -0.006 |  | -0.006 |
|  |  | (0.021) |  | (0.021) |
| =1 if HH owns a cell phone |  | -0.022 |  | -0.022 |
|  |  | (0.045) |  | (0.044) |
| Education level (# Years) |  | -0.007 |  | -0.007 |
|  |  | (0.009) |  | (0.009) |
| Number of children in school |  | -0.031* |  | -0.031* |
|  |  | (0.017) |  | (0.017) |
| =1 if input dealer is in village |  | 0.344*** |  | 0.343** |
|  |  | (0.133) |  | (0.134) |
| =1 if extension agent is in village |  | 0.013 |  | 0.012 |
|  |  | (0.036) |  | (0.036) |
| =1 if HH bought certified chemical |  | 0.183** |  | 0.184** |
|  |  | (0.076) |  | (0.076) |
| Distance from the main market (Km) |  | -0.002 |  | -0.002 |
|  |  | (0.005) |  | (0.005) |
| Savings at the start of harvest season (x 1,000 F CFA) |  | -2E-06 |  | -1.4E-06 |
|  |  | (2E-05) |  | (2E-05) |
| Farm size (Ha) |  | -0.004 |  | -0.004 |
|  |  | (0.003) |  | (0.003) |
| Age of household's head |  | -0.001 |  | -0.001 |
|  |  | (0.001) |  | (0.001) |
| =1 if household’s head is male |  | 0.158** |  | 0.157** |
|  |  | (0.063) |  | (0.063) |
| Household size |  | 0.002 |  | 0.002 |
|  |  | (0.004) |  | (0.004) |
| =1 if HH uses more than one container |  | -0.046 |  | -0.046 |
|  |  | (0.038) |  | (0.039) |
| Dummies for types of containers included |  | Yes |  | Yes |
| Village dummies included |  | Yes |  | Yes |
| Observations | 707 | 707 | 707 | 707 |
| Pseudo R2 | 0.162 | 0.349 | 0.163 | 0.349 |

***Notes***: Results are obtained from probit combined with Mundlack-Chamberlin (MC) device; all predictors are Average Partial Effect (APE), standard errors in parentheses; estimates are obtained using the Stata margin commands, and for the interaction term they are similar to those obtained with the command inteff (Norton *et al*., 2004); *** *p*<0.01, ** *p*<0.05, * *p*<0.1;^a^ In column (4), joint F-test in column (4) is less than 0.01; average effects are also included; US$ 1.00 = 512 F CFA at the time of the survey.

**Appendix F2.** Factors that affect the quantity of maize sold in post-harvest season (OLS-MC)

| **Dependent variable = Log (Quantity sold)** | **(1)** | **(2)** | **(3)** | **(4)** |
| --- | --- | --- | --- | --- |
| Expenditures on insecticides (x 1,000 F CFA) | 0.048*** | 0.058*** | 0.029*** | 0.041*** |
|  | (0.016) | (0.014) | (0.010) | (0.009) |
| =1 HH perceives a risk of food safety from chemical | 0.063 | -0.008 | -0.010 | -0.066 |
|  | (0.104) | (0.106) | (0.112) | (0.111) |
| Expenditures on insecticides x (=1 HH perceives risk) |  |  | 0.061*** | 0.055*** |
|  |  |  | (0.013) | (0.014) |
| =1 if HH stores for sale only | 0.305 | 0.442 | 0.241 | 0.371 |
|  | (0.741) | (0.663) | (0.722) | (0.650) |
| =1 if HH stores for consumption & sale | -0.259 | -0.181 | -0.271 | -0.194 |
|  | (0.423) | (0.392) | (0.423) | (0.397) |
| =1 HH uses more than one container | 0.362* | 0.466** | 0.361* | 0.460** |
|  | (0.210) | (0.182) | (0.210) | (0.182) |
| Quantity of maize stored (Kg) | 2E-04*** | 2E-04*** | 2E-04*** | 2E-04*** |
|  | (3E-05) | (3E-05) | (3E-05) | (3E-05) |
| if HH bought certified chemical | -0.558* | -0.789*** | -0.563* | -0.779*** |
|  | (0.295) | (0.259) | (0.307) | (0.261) |
| =1 if HH reports a case of chemical intoxication in village |  | 0.655 |  | 0.709 |
|  |  | (0.509) |  | (0.525) |
| HH has received information about chemical (# Years) |  | -0.234* |  | -0.232* |
|  |  | (0.127) |  | (0.133) |
| HH owns a radio (# Years) |  | -0.278** |  | -0.276** |
|  |  | (0.128) |  | (0.128) |
| HH owns a TV (# Years) |  | -0.049 |  | -0.020 |
|  |  | (0.105) |  | (0.105) |
| =1 if HH owns a cell phone |  | 0.370* |  | 0.365* |
|  |  | (0.209) |  | (0.209) |
| Education level (# Years) |  | -0.038 |  | -0.034 |
|  |  | (0.024) |  | (0.024) |
| Number of children in school |  | 0.109 |  | 0.097 |
|  |  | (0.077) |  | (0.077) |
| =1 if input dealer is in village |  | -0.229 |  | -0.236 |
|  |  | (0.240) |  | (0.242) |
| =1 if extension agent is in village |  | -0.023 |  | -0.013 |
|  |  | (0.148) |  | (0.146) |
| Post-harvest price (F CFA/Kg) |  | 0.003 |  | 0.003 |
|  |  | (0.002) |  | (0.002) |
| Distance from the main market (Km) |  | 0.004 |  | 0.004 |
|  |  | (0.016) |  | (0.016) |
| Savings at the start of harvest season (x 1,000 F CFA) |  | 3E-04 |  | 3E-04 |
|  |  | (4E-04) |  | (4E-04) |
| Farm size (Ha) |  | 0.024** |  | 0.025** |
|  |  | (0.011) |  | (0.011) |
| Age of household's head |  | -0.001 |  | -0.002 |
|  |  | (0.005) |  | (0.005) |
| =1 if HH's head is male |  | 0.510*** |  | 0.486*** |
|  |  | (0.185) |  | (0.185) |
| Household size |  | -0.004 |  | -0.004 |
|  |  | (0.021) |  | (0.021) |
| Constant | 5.672*** | 4.540*** | 5.698*** | 4.614*** |
|  | (0.234) | (0.573) | (0.236) | (0.579) |
| Observations | 432 | 432 | 432 | 432 |
| Adjusted R-squared | 0.702 | 0.732 | 0.707 | 0.736 |

***Notes***: Standard errors in parentheses; *** *p*<0.01, ** *p*<0.05, * *p*<0.1; village dummies, time and average effects are included; US$ 1.00 = 512 F CFA at the time of the survey.
